# Interaction between Coffee Drinking and TRIB1 rs17321515 Single Nucleotide Polymorphism on Coronary Heart Disease in a Taiwanese Population

**DOI:** 10.3390/nu12051301

**Published:** 2020-05-02

**Authors:** Yin-Tso Liu, Disline Manli Tantoh, Lee Wang, Oswald Ndi Nfor, Shu-Yi Hsu, Chien-Chang Ho, Chia-Chi Lung, Horng-Rong Chang, Yung-Po Liaw

**Affiliations:** 1 Institute of Medicine, Chung Shan Medical University, Taichung City 40201, Taiwan; cvsliu0325@gmail.com; 2 Department of Cardiovascular Surgery, Asia University Hospital, Taichung 41354, Taiwan; 3 Department of Medical Imaging, Chung Shan Medical University Hospital, Taichung City 40201, Taiwan; tantohdisline@gmail.com; 4 Department of Public Health and Institute of Public Health, Chung Shan Medical University, Taichung 40201, Taiwan; wl@csmu.edu.tw (L.W.); nforoswald22@gmail.com (O.N.N.); sui0209@gmail.com (S.-Y.H.); dinoljc@csmu.edu.tw (C.-C.L.); 5 Department of Physical Education, Fu Jen Catholic University, New Taipei 24205, Taiwan; ccho1980@gmail.com; 6 Research and Development Center for Physical Education, Health, and Information Technology, Fu Jen Catholic University, New Taipei 24205, Taiwan; 7 Division of Nephrology, Department of Internal Medicine, Chung Shan Medical University Hospital, Taichung 40201, Taiwan; 8 School of Medicine, Chung Shan Medical University, Taichung 40201, Taiwan

**Keywords:** coffee drinking, TRIB1, rs17321515, CHD, Taiwan Biobank

## Abstract

A complex interplay of several genetic and lifestyle factors influence coronary heart disease (CHD). We determined the interaction between coffee consumption and the *tribbles pseudokinase 1* (*TRIB1*) rs17321515 variant on coronary heart disease (CHD). Data on CHD were obtained from the National Health Insurance Research Database (NHIRD) while genotype data were collected from the Taiwan Biobank (TWB) Database. From the linked electronic health record data, 1116 individuals were identified with CHD while 7853 were control individuals. Coffee consumption was associated with a lower risk of CHD. The multivariate-adjusted odds ratio (OR) and 95% confidence interval (CI) was 0.84 (0.72–0.99). Association of CHD with the *TRIB1* rs17321515 variant was not significant. The OR (95% CI) was 1.01 (0.72–0.99). There was an interaction between *TRIB1* rs17321515 and coffee consumption on CHD risk (*p* for interaction = 0.0330). After stratification by rs17321515 genotypes, coffee drinking remained significantly associated with a lower risk of CHD only among participants with GG genotype (OR, 0.62; 95% CI, 0.45–0.85). In conclusion, consumption of coffee was significantly associated with a decreased risk of CHD among Taiwanese adults with the *TRIB1* GG genotype.

## 1. Introduction

Coronary heart disease, also known as ischemic heart disease (IHD) or coronary artery disease (CAD) is the top cause of global mortality [1,2]. It remains the second leading cause of death in Taiwan [3]. The global coronary heart disease (CHD) mortality is projected to grow from 7.594 million in 2016 to about 9.245 million in 2030 [4].

A complex interplay of numerous genetic and lifestyle factors influence the onset of CHD [5,6,7]. Genotypes are nonmodifiable factors, so they cannot be confounded by other factors. As such, they are capable of playing direct causal roles in disease development [8,9]. Identification of genetic variants associated with diseases and the underlying pathophysiological mechanisms is an important step in the development of potential drug targets [8].

Genetic predisposition accounts for about 30%–60% of CHD [10,11]. Despite this, most underlying genes and molecular pathways are yet to be fully explored and therefore a significant portion of CHD heritability is not clearly understood [2]. For instance, SNPs account for just a minute fraction (approximately 10–15%) of CHD heritability [1,2,5,12,13]. The *TRIB1* is among the top genes having genome-wide significant single nucleotide polymorphisms (SNPs) for CHD [14]. It is located on chromosome 8q24 and is greatly involved in cholesterol metabolism and atherosclerosis process [15]. One of its variants, rs17321515, has been associated with variations in plasma lipid levels and CHD [14,16,17,18].

Coffee is a popular beverage that is widely consumed in the world [19]. In Taiwan, coffee consumption has grown rapidly in recent years. So far, the local coffee industry has expanded significantly [20]. Several studies have investigated the effects of coffee consumption on CHD. However, results have been controversial. For instance, in one of the studies, excessive consumption was significantly associated with a moderate increase in the risk of CHD [21]. However, in another study, CHD risk was higher among moderate than for excessive coffee consumers [22]. Cardioprotective effects of coffee may stem from its richness in bioactive compounds like polyphenols that possess hypocholesterolemic, antihypertensive, anti-inflammatory, and antioxidant properties [23,24]. The antioxidant content in coffee was found to be higher than that in tea, vegetables, and fruits [25].

It is well known that interactions between genes and the environment influence disease outcomes [26]. So far, there is substantial information on genetic variation and dietary patterns (including but not limited to coffee consumption) and the risk of CHD. Results from a previous study indicated that a variant in the *cytochrome P450 1A2 gene (CYP1A2)* modifies the association between caffeinated coffee consumption and the risk of myocardial infection [27]. Nevertheless, pinpointing a specific polymorphic variant is challenging considering that individual differences may exist in response to coffee or caffeine. To our knowledge, no prior study has discussed specific genotypes that can modify the association between coffee intake and the risk of CHD in Taiwan. In light of this, we determined the interaction between coffee consumption and the *TRIB1* rs17321515 variant on CHD.

## 2. Materials and Methods

### 2.1. Data Source and Participants

We used electronic data of Taiwan Biobank (TWB) participants recruited between 2008 and 2015. Participants provided blood samples for DNA extraction and completed questionnaires covering a wide range of medical, social, and lifestyle information. All participants provided informed consent. Genotyping was done using the Axiom™ Genome-Wide TWB 2.0 Array plate (Santa Clara, CA, USA). Data on CHD between 1998 and 2015 were obtained from the National Health Insurance Research Database (NHIRD). The TWB database was linked to the NHIRD using encrypted personal identification numbers. This study was approved by the Institutional Review Board of Chung Shan Medical University (CS2-16114).

In total, 9001 biobank participants were recruited. After excluding persons with incomplete questionnaires (*n* = 13) and genotype information (*n* = 19), 1116 coronary heart disease patients and 7853 controls were included in the study.

### 2.2. Assessment of Variables

Coronary heart disease was identified based on either two outpatient visits or one admission with reported International Classification of Diseases, Ninth Revision, Clinical Modification (ICD-9-CM) code 410–414. Participants were classified as regular coffee drinkers if they drank coffee at least three days per week in the last 6 months. Details of the covariates and physical measures used in the text have been described in our recent publication [28].

### 2.3. Selection of the Polymorphic Variant

The rs17321515 variant in the *TRIB1* gene was selected based on the literature search. This variant was selected because of its previous associations with CHD and dyslipidemia, especially in Han Chinese populations [16,17]. We also searched Google Scholar and selected rs762551 variant in the CYP1A2 gene which has been associated with caffeine metabolism and increased risk of myocardial infarction. We followed a standard quality control procedure and excluded SNPs with (1) a low call rate (<95%), (2) *p*-value of <1.0 × 10^−3^ for the Hardy–Weinberg equilibrium test, and (3) minor allele frequency of <0.05. Moreover, we removed one individual from the pair of related samples based on pairwise identity-by-descent (IBD).

### 2.4. Statistical Analysis

We used the statistical analysis system (SAS) software (version 9.4, SAS Institute, Cary, NC, USA) and PLINK (v1.09, http://pngu.mgh.harvard.edu/purcell/plink/) to perform analyses. Differences between groups were compared using the chi-square test. Associations of coffee and the rs17321515 variant with CHD were determined using logistic regression analysis. Adjusted variables included sex, age, educational level, smoking, alcohol intake, tea consumption, vegetarian diet, body mass index (BMI), diabetes, hypertension, hyperlipidemia, atrial fibrillation, and *CYP1A2* rs762551 variant. Odds ratios with their 95% confidence intervals were estimated.

## 3. Results

The descriptive data of 1116 participants with CHD and 7863 control individuals are shown in Table 1. Significant differences existed between patients and controls for coffee drinking, sex, age, educational level, cigarette smoking, exercise, body mass index (BMI), diabetes, hypertension, hyperlipidemia, atrial fibrillation, and vegetarian diet (*p* < 0.05). However, there were no significant differences between patients and controls for the *TRIB1* rs17321515 and *CYP1A2* rs762551 genotypes, alcohol, and tea consumption. Differences in coffee consumption habits between men and women as well as between those in different age groups are shown in Table 2.

Coffee drinking was associated with a lower risk of CHD (OR, 0.84; 95% CI, 0.72–0.99), as shown in Table 3. Association with the *TRIB1* rs17321515 variant was not significant; the OR was 1.01, 95% CI = 0.87–1.18. However, for the *CYP1A2* rs762551 variant, the OR was 0.86 with a 95% CI of 0.74–0.99 for AC+CC, compared to the AA genotype. Corresponding ORs (95% CI) for CHD were 1.53 (1.07–2.19) for ages 40–49 years, 3.92 (2.82–5.46) for ages 50–59 years, 6.46 (4.59–9.09) for ages 60–70 years, 1.23 (1.04–1.46) for overweight, 1.35 (1.11–1.63) for obesity, 1.19 (1.01–1.41) for diabetes, 3.40 (2.91–3.98) for hypertension, 2.25 (1.91–2.63) for hyperlipidemia, and 4.09 (2.14–7.82) for atrial fibrillation.

There was a significant interaction (*p* = 0.0330) between *TRIB1* rs17321515 and coffee drinking on CHD risk (Table 4). After stratification by rs17321515 genotypes, coffee drinking remained significantly associated with a lower risk of CHD only among those with the GG genotype (OR, 0.62; 95% CI, 0.45–0.85). There was no interaction between the *CYP1A2* rs762551 variant and coffee consumption.

## 4. Discussion

In the current study, we determined whether an interactive association exists between coffee intake and the *TRIB1* rs17321515 variant with the risk of CHD. Our findings offered unique evidence that coffee intake might have a protective effect on CHD. We also found that contrary to previous findings [17,29], rs17321515 was not associated with CHD. Importantly, we found evidence of an interaction between rs17321515 and coffee intake. After stratification by rs17321515 genotypes, we found that CHD risk was significantly lower among those with GG genotype who consumed coffee relative to their non-coffee-drinking counterparts. However, there was no association among those with the GA+AA genotype, indicating that the genotype may not have any effect on CHD. *TRIB1* rs17321515 has been associated with a decreased risk of CAD among Europeans, Malays, and Asian Indians [15,30,31]. However, their analyses were not performed based on coffee intake.

So far, several studies have investigated the independent effects of coffee intake and *TRIB1* rs17321515 on cardiovascular disease risk. Of the studies, those investigating coffee consumption and cardiovascular disease risk have shown conflicting results. Contrary to findings from case–control studies which suggested that coffee intake was detrimental to coronary arteries [32], umbrella reviews of observational and intervention studies have found it to be beneficial even in little amounts [33,34]. An increased risk of CHD previously reported among heavy coffee drinkers was attributed to smoking [35]. In light of this, we included smoking in our analysis.

Regarding the rs17321515 polymorphism, its AA+GA genotypes were previously associated with an increased risk of CHD among Han Chinese [36]. In a Singapore Malay Eye study of 3280 adults aged 40–79 years old, the odds ratio for CHD among carriers of this variant was 1.23 for each copy of the A allele [31]. Even though the rs17321515 variant has been assessed in Asian populations as noted above, attempts have not been made to replicate it in Taiwan. This was the motivation behind the selection of this variant for the current study.

As stated earlier, lifestyle changes and genetic factors play a substantial role in the development of cardiovascular diseases. Of note, the interactive associations of both factors with CHD have not been widely reported. When coffee intake and the *TRIB1* rs17321515 variant were included in our model with adjustments for smoking and other lifestyle variables, we found that the GG genotype was significantly protective against CHD disease in individuals who consumed coffee compared to those who did not. The underlying mechanisms of interaction between coffee drinking and *TRIB1* rs17321515 SNP on CHD are not completely understood. However, metabolites in coffee are believed to influence protective endogenous pathways by modulation of gene expression [37].

One of the main variables included in our model was the rs762551 variant in the *CYP1A2* gene. We chose this variant based on its previous association with caffeine metabolism and its role in modifying the association between caffeinated coffee and the risk of heart disease [27]. Contrary to expectation, we found that AC+CC, compared to the AA genotype was protective against CHD in both the adjusted (OR, 0.86; 95% CI (0.74–0.99) and the separate model (Supplementary Table S1). By performing stratified analyses, we found that associations of *CYP1A2* rs762551 genotypes with CHD were not significant (Supplementary Table S2). Besides, there was no interaction between the variant and coffee consumption. Given that our findings are based on a limited number of coffee consumers, further investigations would be needed to clarify these associations.

In this study, we also observed that coffee consumption habits between cases and controls differed significantly based on gender and different age groups. However, differences in consumption based on gender and age are yet to be adequately determined, particularly in Taiwan.

We believe that these results will help to enhance the knowledge on the role of coffee in the association between rs17321515 variant and CHD among Taiwanese adults. However, the current study is just a first step to examine this association, which remains a fundamental issue for future research.

This study was limited in several ways. First, about 70% of the population studied did not consume any coffee. Such a limited number of coffee drinkers may preclude the possibility of observing meaningful associations between coffee and CHD. Next, our questionnaire did not have information on the type of coffee, caffeine content (that is, caffeinated or decaffeinated), methods of preparation, and the daily amount of consumption. We understand that these attributes may have different effects on CHD. Therefore, we recommend further research in this area. Second, well-established risk factors such as smoking, exercise, education, male sex, diabetes, tea-drinking, and vegetarian diet were not associated with the risk of CHD in the current population. This is an indication that our study population might not be representative of typical CHD study populations. Third, there is a possibility of nondifferential misclassification bias as information on coffee intake was based on self-report Lastly, even though the TWB is representative of the general population, only individuals who are 30–70 years old were recruited in the project. Therefore, we could not analyze data of adults under 30 or over 70 years of age.

## 5. Conclusions

In conclusion, our findings highlight the interactive association of coffee drinking and *TRIB1* rs17321515 polymorphism on coronary heart disease in Taiwanese adults. Taken together, we found that the risk of CHD was significantly lower among those with GG genotype who consumed coffee compared to their non-coffee-drinking counterparts. These results have provided considerable knowledge on gene–nutrient interaction in relation to cardiovascular disease.

## Figures and Tables

**Table 1:** Descriptive data of the study participants.

Variable	Controls (*n* = 7853)	CHD Patients (*n* = 1116)	*p*-Value
	*n* (%)	*n* (%)	
Coffee drinking			<0.0001
No	5269 (67.10)	824 (73.84)	
Yes	2584 (32.90)	292 (26.16)	
*TRIB1* rs17321515			0.9920
GG	2362 (30.08)	335 (30.02)	
GA+AA	5491 (69.92)	781 (69.98)	
*CYP1A2* rs762551			0.1490
AA	3326 (42.35)	500 (44.80)	
AC+CC	4527 (57.65)	616 (55.20)	
Sex			<0.0001
Women	4275 (54.44)	520 (46.59)	
Men	3578 (45.56)	596 (53.41)	
Age (years)			<0.0001
30–39	2042 (26.00)	46 (4.12)	
40–49	2337 (29.76)	111 (9.95)	
50–59	2217 (28.23)	415 (37.19)	
60–70	1257 (16.01)	544 (48.75)	
Educational level			<0.0001
Elementary school	493 (6.28)	170 (15.23)	
Junior and senior high school	3258 (41.49)	498 (44.62)	
University and above	4102 (52.23)	448 (40.14)	
Cigarette smoking			0.0060
No	6117 (77.89)	828 (74.19)	
Yes	1736 (22.11)	288 (25.81)	
Alcohol drinking			0.3540
No	7031 (89.53)	989 (88.62)	
Yes	822 (10.47)	127 (11.38)	
Exercise			<0.0001
No	4702 (59.88)	474 (42.47)	
Yes	3151 (40.12)	642 (57.53)	
BMI (kg/m^2^)			<0.0001
BMI < 18.5 (Underweight)	215 (2.74)	11 (0.99)	
18.5 ≤ BMI < 24 (Normal weight)	3870 (49.28)	396 (35.48)	
24 ≤ BMI < 27 (Overweight)	2283 (29.07)	415 (37.19)	
BMI ≥ 27 (Obesity)	1485 (18.91)	294 (26.34)	
Diabetes			<0.0001
No	6943 (88.41)	738 (66.13)	
Yes	910 (11.59)	378 (33.87)	
Hypertension			<0.0001
No	6424 (81.80)	391 (35.04)	
Yes	1429 (18.20)	725 (64.96)	
Hyperlipidemia			<0.0001
No	5828 (74.21)	372 (33.33)	
Yes	2025 (25.79)	744 (66.67)	
Atrial fibrillation			<0.0001
No	7833 (99.75)	1089 (97.58)	
Yes	20 (0.25)	27 (2.42)	
Tea consumption			0.1110
No	4894 (62.30)	723 (64.78)	
Yes	2959 (37.68)	393 (35.22)	
Vegetarian diet			0.0090
No	7011 (89.28)	1025 (91.85)	
Yes	842 (10.72)	91 (8.15)	

CHD: Coronary heart disease, BMI: Body mass index, TRIB1: tribbles pseudokinase 1; CYP1A2: cytochrome P450 1A2. GG, GA, and AA represent genotypes in the TRIB1 rs17321515 variant while AA, AC, and CC represent genotypes in the CYP1A2 rs762551 variant.

**Table 2:** Characteristics of study participants based on coffee consumption.

	No Coffee Drinking				Coffee Drinking				*p*-Value
	Controls		CHD Patients		Controls		CHD Patients		
	*n*	%	*n*	%	*n*	%	*n*	%	
*TRIB1* rs17321515									0.4130
GG	1574	29.87	261	31.67	788	30.50	74	25.34	
GA	2564	48.66	396	48.06	1272	49.23	148	50.68	
AA	1131	21.47	167	20.27	524	20.28	70	23.97	
*CYP1A2* rs762551									0.5160
AA	2229	42.30	361	43.81	1097	42.45	139	47.60	
AC	2411	45.76	375	45.51	1176	45.51	126	43.15	
CC	629	11.94	88	10.68	311	12.04	27	9.25	
Sex									<0.0001
Women	2785	52.86	391	47.45	1490	57.66	129	44.18	
Men	2484	47.14	433	52.55	1094	42.34	163	55.82	
Age									<0.0001
30–39	1340	25.43	31	3.76	702	27.17	15	5.14	
40–49	1485	28.18	77	9.34	852	32.97	34	11.64	
50–59	1521	28.87	310	37.62	696	26.93	105	35.96	
60–70	923	17.52	406	49.27	334	12.93	138	47.26	
Education									<0.0001
Elementary school	379	7.19	143	17.35	114	4.41	27	9.25	
Junior and Senior high school	2259	42.87	375	45.51	999	38.66	123	42.12	
University and above	2631	49.93	306	37.14	1471	56.93	142	48.63	
Cigarette smoking									<0.0001
No	4174	79.22	628	76.21	1943	75.19	200	68.49	
Yes	1095	20.78	196	23.79	641	24.81	92	31.51	
Alcohol drinking									0.1890
No	4734	89.85	737	89.44	2297	88.89	252	86.30	
Yes	535	10.15	87	10.56	287	11.11	40	13.70	
Physical activity									<0.0001
No	3142	59.63	353	42.84	1560	60.37	121	41.44	
Yes	2127	40.37	471	57.16	1024	39.63	171	58.56	
BMI (kg/m^2^)									<0.0001
BMI < 18.5	156	2.96	9	1.09	59	2.28	2	0.68	
18.5 ≤ BMI < 24	2629	49.90	308	37.38	1241	48.03	88	30.14	
24 ≤ BMI < 27	1491	28.30	294	35.68	792	30.65	121	41.44	
BMI ≥ 27	993	18.85	213	25.85	492	19.04	81	27.74	
Diabetes									<0.0001
No	4631	87.89	538	65.29	2312	89.47	200	68.49	
Yes	638	12.11	286	34.71	272	10.53	92	31.51	
Hypertension									<0.0001
No	4237	80.41	280	33.98	2187	84.64	111	38.01	
Yes	1032	19.59	544	66.02	397	15.36	181	61.99	
Hyperlipidemia									<0.0001
No	3873	73.51	285	34.59	1955	75.66	87	29.79	
Yes	1396	26.49	539	65.41	629	24.34	205	70.21	
Atrial fibrillation									<0.0001
No	5255	99.73	804	97.57	2578	99.77	285	97.60	
Yes	14	0.27	20	2.43	6	0.23	7	2.40	
Tea consumption									<0.0001
No	3518	66.77	571	69.3	1376	53.25	152	52.05	
Yes	1751	33.23	253	30.7	1208	46.75	140	47.95	
Vegetarian diet									<0.0001
No	4646	88.18	761	92.35	2365	91.52	264	90.41	
Yes	623	11.82	63	7.65	219	8.48	28	9.59	

CHD: Coronary heart disease, BMI: Body mass index, *TRIB1: tribbles pseudokinase 1, CYP1A2: cytochrome P450 1A2*.

**Table 3:** Association of CHD with associated variables.

Variable	OR	95% CI
Coffee drinking (ref: No)		
Yes	0.84	0.72–0.99
*TRIB1* rs17321515 (ref: GG)		
GA+AA	1.01	0.87–1.18
*CYP1A2* rs762551 (ref: AA)		
AC+CC	0.86	0.74–0.99
Sex (ref: Women)		
Men	1.17	0.98–1.39
Age (ref: 30–39)		
40–49	1.53	1.07–2.19
50–59	3.92	2.82–5.46
60–70	6.46	4.59–9.09
Educational level (ref: Elementary school)		
Junior and senior high school	0.97	0.77–1.21
University and above	1.01	0.80–1.28
Cigarette smoking (ref: No)		
Yes	1.07	0.88–1.30
Alcohol drinking (ref: No)		
Yes	0.79	0.62–1.01
Exercise (ref: No)		
Yes	1.07	0.92–1.24
BMI (ref: 18.5 ≤ BMI < 24)		
BMI < 18.5	0.78	0.40–1.51
24 ≤ BMI < 27	1.23	1.04–1.46
BMI ≥ 27	1.35	1.11–1.63
Diabetes (ref: No)		
Yes	1.19	1.01–1.41
Hypertension (ref: No)		
Yes	3.40	2.91–3.98
Hyperlipidemia (ref: No)		
Yes	2.25	1.91–2.63
Atrial fibrillation (ref: No)		
Yes	4.09	2.14–7.82
Tea consumption (ref: No)		
Yes	0.97	0.83–1.13
Vegetarian diet (ref: No)		
Yes	0.96	0.75–1.24

Ref: reference, CHD: Coronary heart disease, BMI: Body mass index, OR: odds ratio, CI: confidence interval, *TRIB1: tribbles pseudokinase 1, CYP1A2: cytochrome P450 1A2*.

**Table 4:** Association of CHD with coffee drinking stratified by rs17321515 genotypes.

Variable	*TRIB1* rs17321515 (GG)		*TRIB1* rs17321515 (GA+AA)	
	OR	95% CI	OR	95% CI
Coffee drinking (ref: No)				
Yes	0.62	0.45–0.85	0.95	0.79–1.15
*CYP1A2* rs762551 (ref: AA)				
AC+CC	0.83	0.64–1.08	0.86	0.72–1.02
Sex (ref: Women)				
Men	1.26	0.91–1.74	1.13	0.92–1.38
Age (ref: 30–39)				
40–49	0.79	0.41–1.54	2.01	1.30–3.10
50–59	3.46	1.96–6.12	4.21	2.79–6.35
60–70	5.52	3.05–10.00	7.10	4.66–10.84
Educational level (ref: Elementary school)				
Junior and senior high school	1.16	0.76–1.77	0.91	0.69–1.18
University and above	1.22	0.78–1.89	0.94	0.71–1.25
Cigarette smoking (ref: No)				
Yes	0.90	0.63–1.30	1.15	0.91–1.45
Alcohol drinking (ref: No)				
Yes	0.74	0.47–1.17	0.81	0.61–1.08
Exercise (ref: No)				
Yes	1.04	0.79–1.36	1.08	0.90–1.29
BMI (ref: 18.5 ≤ BMI < 24)				
BMI < 18.5	1.91	0.68–5.40	0.51	0.21–1.21
24 ≤ BMI < 27	1.49	1.09–2.05	1.15	0.94–1.40
BMI ≥ 27	2.03	1.43–2.88	1.14	0.90–1.43
Diabetes (ref: No)				
Yes	1.12	0.82–1.53	1.22	1.00–1.49
Hypertension (ref: No)				
Yes	3.84	2.87–5.12	3.28	2.72–3.96
Hyperlipidemia (ref: No)				
Yes	1.94	1.44–2.60	2.40	1.99–2.90
Atrial fibrillation (ref: No)				
Yes	8.13	2.44–27.09	3.10	1.42–6.77
Tea consumption (ref: No)				
Yes	1.14	0.86–1.52	0.91	0.75–1.09
Vegetarian diet (ref: No)				
Yes	0.88	0.54–1.42	0.99	0.74–1.33
rs17321515*coffee	*p* = 0.0330			

Ref: reference, CHD: Coronary heart disease, BMI: Body mass index, OR: odds ratio, CI: confidence interval, *TRIB1: tribbles pseudokinase 1, CYP1A2: cytochrome P450 1A2*.

## Supplementary Materials

The following are available online at https://www.mdpi.com/2072-6643/12/5/1301/s1. Table S1: Association of CHD with rs762551 variant and associated factors, Table S2: Association of CHD with coffee drinking stratified by rs762551 genotypes.

## Abbreviations

SNP: single nucleotide polymorphism, CHD: coronary heart disease, TWB: Taiwan Biobank, NHIRD: National Health Insurance Research Database, OR: odds ratio, CI: confidence interval, BMI: body mass index, *ICD*-*9*-*CM:* International Classification of Diseases, Ninth Revision, Clinical Modification.
