# Supplementary material for: Interaction between Coffee Drinking and TRIB1 rs17321515 Single Nucleotide Polymorphism on Coronary Heart Disease in a Taiwanese Population
Source: Nutrients. 2020 May 2;12(5):1301. doi: 10.3390/nu12051301 (PMC7285234; doi:10.3390/nu12051301)
Supplement: Supplementary file 1 [file nutrients-12-01301-s001.pdf]

nutrients

MDPI

Supplementary

Table S1. Association of CHD with rs762551 variant and associated factors.

	OR	95% CI		
Coffee drinking (ref: No)				
Yes	0.84	0.72	-	0.99
CYP1A2 rs762551(ref:AA)				
AC+CC	0.86	0.74	-	0.99
Sex (ref: Women)				
Men	1.17	0.98	-	1.39
Age (ref:30-39)				
40-49	1.53	1.07	-	2.19
50-59	3.92	2.81	-	5.46
60-70	6.46	4.59	-	9.09
Education (ref: Elementary school)				
Junior and Senior high school	0.97	0.77	-	1.21
University above	1.01	0.80	-	1.28
Cigarette Smoking (ref: No)				
Yes	1.07	0.88	-	1.30
Alcohol drinking (ref: No)				
Yes	0.79	0.62	-	1.01
Physical activity (ref: No)				
Yes	1.07	0.92	-	1.24
BMI (ref: 18.5 ≤ BMI < 24)				
BMI < 18.5	0.78	0.40	-	1.51
24 ≤ BMI < 27	1.23	1.04	-	1.46
BMI ≥ 27	1.35	1.11	-	1.63
Diabetes (ref: No)				
Yes	1.19	1.01	-	1.41
Hypertension (ref: No)				
Yes	3.40	2.91	-	3.98
Hyperlipidemia (ref: No)				
Yes	2.25	1.92	-	2.63
Atrial fibrillation (ref: No)				
Yes	4.09	2.14	-	7.82
Tea consumption (ref: No)				
Yes	0.97	0.83	-	1.13
Vegetarian diet (ref: No)				
Yes	0.96	0.75	-	1.24

Ref: reference, CHD: Coronary heart disease, BMI: Body mass index, OR: odds ratio, CI: confidence interval, CYP1A2: cytochrome P450 1A2.

Table S2. Association of CHD with coffee drinking stratified by rs762551 genotypes.

	CYP1A2 rs762551 (AA)				CYP1A2 rs762551 (AC+CC)			
	OR	95% CI			OR	95% CI		
Coffee drinking (ref: No)								
Yes	0.84	0.66	-	1.06	0.85	0.68	-	1.06
Sex (ref: Women)								
Men	1.20	0.92	-	1.56	1.15	0.91	-	1.44
Age (ref:30-39)								
40-49	1.33	0.80	-	2.20	1.77	1.06	-	2.95
50-59	3.18	1.99	-	5.07	4.82	3.00	-	7.72
60-70	4.89	3.01	-	7.94	8.30	5.12	-	13.48
Education (ref: Elementary school)								
Junior and Senior high school	1.02	0.72	-	1.45	0.93	0.69	-	1.24
University above	1.00	0.69	-	1.44	1.03	0.75	-	1.40
Cigarette smoking (ref: No)								
Yes	1.09	0.82	-	1.45	1.05	0.80	-	1.37
Alcohol drinking (ref: No)								
Yes	1.04	0.74	-	1.46	0.61	0.43	-	0.86
Physical activity (ref: No)								
Yes	1.22	0.98	-	1.52	0.96	0.79	-	1.18
BMI (ref: 18.5 ≤ BMI < 24)								
BMI < 18.5	0.40	0.12	-	1.36	1.20	0.54	-	2.65
24 ≤ BMI < 27	1.27	0.98	-	1.63	1.22	0.97	-	1.53
BMI ≥ 27	1.44	1.08	-	1.91	1.28	0.99	-	1.66
Diabetes (ref: No)								
Yes	1.17	0.91	-	1.51	1.21	0.96	-	1.51
Hypertension (ref: No)								
Yes	3.41	2.70	-	4.31	3.43	2.77	-	4.23
Hyperlipidemia (ref: No)								
Yes	2.33	1.84	-	2.94	2.22	1.79	-	2.75
Atrial fibrillation (ref: No)								
Yes	3.08	0.98	-	9.65	5.05	2.26	-	11.27
Tea consumption (ref: No)								
Yes	1.05	0.83	-	1.31	0.91	0.73	-	1.12
Vegetarian diet (ref: No)								
Yes	0.79	0.54	-	1.17	1.14	0.82	-	1.59
rs762551*coffee drinking	$p = 0.8295$							

Ref: reference, CHD: Coronary heart disease, BMI: Body mass index, OR: odds ratio, CI: confidence interval, CYP1A2: cytochrome P450 1A2.
